# Differentiating Desquamating Skin Lesions: A Case of Methotrexate Epidermal Necrosis

**DOI:** 10.7759/cureus.50050

**Published:** 2023-12-06

**Authors:** Sahifah Ansari, Dina H Zamil, Edgar Rodriguez, Carly Dunn, Soo Jung Kim

**Affiliations:** 1 Dermatology, Baylor College of Medicine, Houston, USA

**Keywords:** stevens-johnson syndrome, necrotizing fasciitis, methotrexate toxicity, desquamating skin lesions, adverse drug reactions

## Abstract

Desquamating skin lesions are a non-specific finding that requires urgent evaluation given the life-threatening severity of one of the potential causes, Stevens-Johnson syndrome (SJS). Methotrexate toxicity, also known in its cutaneous form as methotrexate epidermal necrosis (MEN), is another entity that presents similarly to SJS and is described here in a patient with increased risk due to his age, chronic kidney disease, and increased dose of methotrexate. His diagnosis was complicated by other historical risk factors, including antibiotic use, but was eventually elucidated when he was noted to have bone marrow suppression. Given the pathophysiology of SJS, a T-cell mediated reaction, the patient’s leukopenia increased the likelihood of MEN as his ultimate diagnosis. However, in light of his aggressive treatment and non-specific histopathology, the clinical suspicion of MEN could not be confirmed.

## Introduction

Desquamating skin lesions can result from a number of conditions, including Stevens-Johnson syndrome/toxic epidermal necrolysis (SJS/TEN), pemphigus vulgaris, bullous pemphigoid, staphylococcal scalded skin syndrome, erythema multiforme major, and generalized fixed drug eruptions [1]. To differentiate such diseases in a patient presenting with symptoms such as conjunctivitis, erythematous macules, and mucosal erosions, urgent histopathology with immunofluorescence is often required due to the immediate danger of SJS/TEN [2]. Although a spectrum of drug toxicities exists and are mostly benign, SJS/TEN represents a life-threatening form of cutaneous drug reaction [3].

Methotrexate, used for inflammatory diseases, can result in several cutaneous reactions, among them SJS/TEN, in addition to maculopapular eruptions and urticaria [4]. As such, it is essential to distinguish cases of methotrexate toxicity from SJS/TEN cases, and when SJS/TEN has resulted from methotrexate itself. Methotrexate affects cell proliferation, leading to mucosal erosions [5]. A number of cases have described methotrexate-associated acral erosions, likely resulting from cytotoxicity to the acral epidermis [4,6].

Methotrexate-induced toxicity is typically thought to be dose-dependent, and patients with kidney disease or an age over 55 may be at increased risk [5]. Here, we present a case of suspected methotrexate toxicity in an elderly male with chronic kidney disease.

This article was previously presented as a poster at the 2023 Annual Rheumatology Nurses Society Conference on August 3, 2023.

## Case presentation

**Figure 1:**
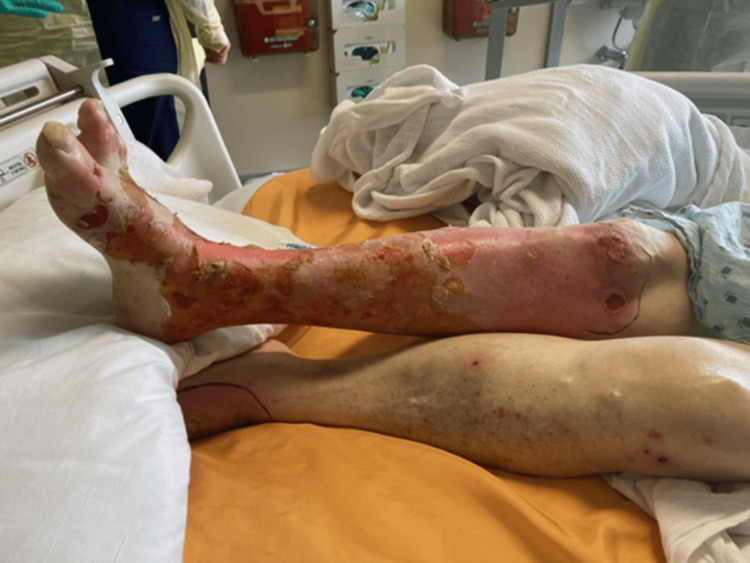
Desquamating skin lesions and blood-crusted erosions on the lower extremities.

The patient is a 74-year-old male with a history of rheumatoid arthritis, coronary artery disease, stage 3A chronic kidney disease (eGFR (estimated glomerular filtration rate) 54.6 mL/min/1.73m^2^), Parkinson’s disease, major depressive disorder, polyneuropathy, and a remote history of seizures. On the first day of the patient’s course, the patient’s primary care physician noted discrete papules on the right lower extremity (RLE) that the patient reported occurred yearly on his legs. At that time, he also complained of a sore throat and was given a 10-day course of amoxicillin. After six days, he presented to the ED for RLE cellulitis after he submerged his leg in a lake to save an alligator. He was treated with one dose each of IV vancomycin and piperacillin-tazobactam and discharged on a 10-day course of clindamycin. He returned to the ED seven days later (15 days from his initial presentation) with desquamating lesions on his RLE and an erythematous, maculopapular rash with blood crusting covering approximately 10% of his total body surface area (Figure 1). He also reported that he began bleeding from his eyes and mouth four days prior to his return to the ED.

**Figure 2:**
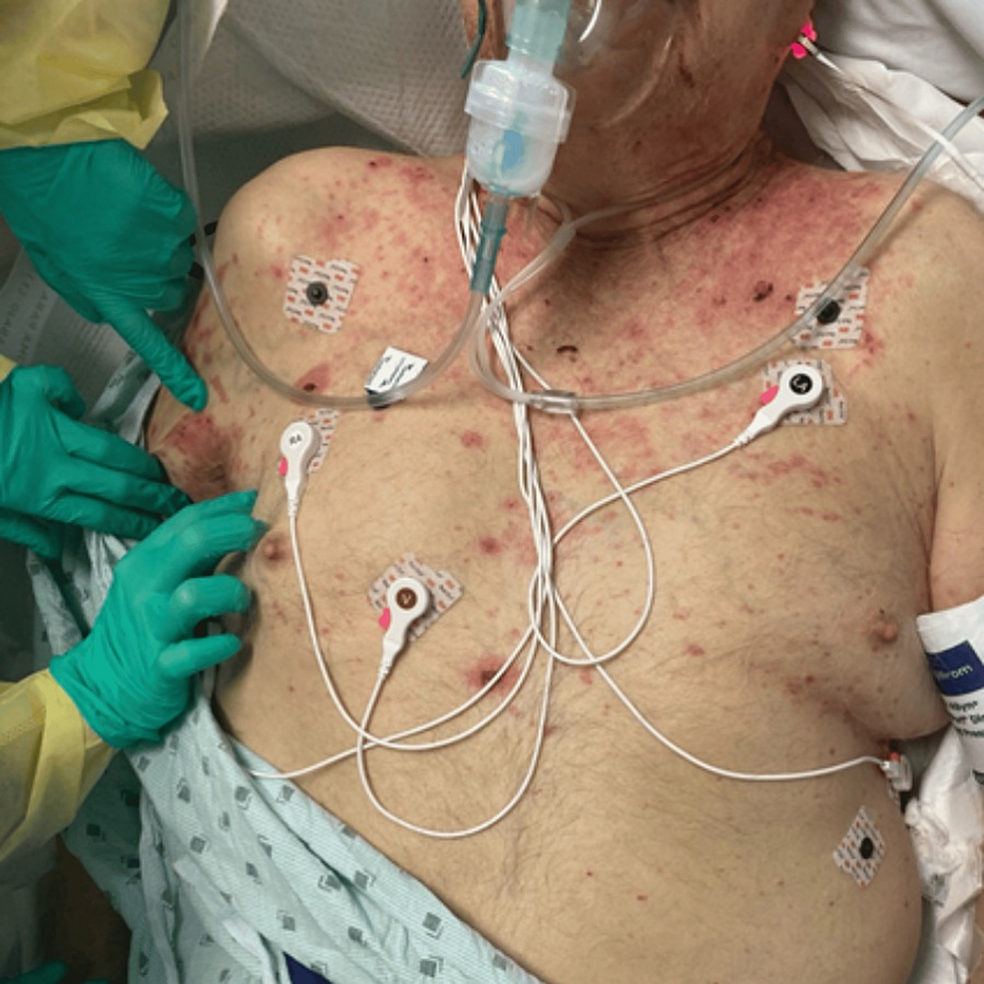
Erythematous macules on patches with areas of confluence.

On exam, the patient was found to have erosions with hemorrhagic crust on the mucosal surfaces of the upper and lower lips and bilateral eyelids without conjunctival or lid-margin involvement. He also had a similar erosion on the hard palate. On the chest, back, and bilateral axillae, he had discrete erythematous macules and patches, specifically with areas of confluence on the chest (Figure 2). Erosions with a violaceous dusky rim were noted on the left dorsal hand and foot, with some bullae on the dorsal fingers of the left hand. The plaque on the RLE extended from the foot to the knee and was edematous, erythematous, and tender to palpation and had large erosions interspersed. Additionally, an erythematous patch was noted on the corona of the penis.

The differential diagnosis in the ED was necrotizing fasciitis versus Stevens-Johnson syndrome (SJS). The patient refused amputation of the RLE. He accepted a single dose of Enbrel (etanercept) for SJS but refused subsequent doses. Empiric treatment for necrotizing fasciitis was started and included aztreonam, doxycycline, linezolid, metronidazole, and moxifloxacin ophthalmic drops (for ocular lesions). A CT scan showed no evidence of necrotizing fasciitis. Blood and tissue cultures were taken and continued to show no growth for 72 hours during his hospital course. He remained afebrile, and his blood pressure was within normal limits. He was tachycardic with his pulse ranging between 110 and 130. His oxygen saturation ranged from 92% to 100% while on two liters of nasal cannula. The vascular surgery team decided that the patient did not need an amputation because they favored the diagnosis of SJS.

However, the patient was also found to have pancytopenia when he was admitted from the ED with a WBC count of 0.2 K/uL, a RBC count of 6.4 x 10^6 K/uL, and a platelet count of 48 K/uL. On chart review, his last known WBC count was 4.6 K/uL a week prior, and it was within normal limits two months earlier. He was given one unit of packed RBCs and one unit of platelets. The next day, his WBC count was still 0.2 K/uL, the RBC count was 8.3 x 10^6 K/uL, and the platelet count was 13 K/uL. Upon further questioning, the team found that the patient was told to double his folate dose by his rheumatologist for his low WBC count, but he accidentally doubled his methotrexate dose instead for about five weeks. He took 30 mg per week instead of the prescribed 15 mg once weekly. Bone marrow biopsy a day after ED admission yielded poor growth despite multiple attempts, indicative of bone marrow failure, and he was diagnosed with severe combined immunodeficiency (SCID). For treatment of his leukopenia, the hematology/oncology team recommended leucovorin and, later, granulocyte colony-stimulating factor (G-CSF) due to minimal improvement on leucovorin alone. A methotrexate level obtained two days later was 0.04 umol/L (the therapeutic level is above 5 umol/L in a 48-hour period). After another two days, the patient’s labs were significant for a platelet count of 6 K/uL and an increased eosinophil differential at 19.6%.

The patient was transferred to a burn facility for more specialized wound care due to the suspicion for SJS. Shortly before transfer, the patient’s sputum cultures returned positive for drug-resistant Candida auris, and he was intubated. At the burn facility, the patient only required supportive treatment for the skin lesions, which resolved during his hospital stay, but he was moved to the ICU for hemodynamic lability and afebrile leukocytosis. His dermatopathology report came back after his transfer, on day 23 of his course. The biopsy showed mild epidermal acanthosis, minimal junctional vacuolar changes, enlarged nuclei with prominent nucleoli, necrotic keratinocytes in superficial layers, multifocal orthokeratosis, and minimal superficial perivascular lymphocytic infiltrates. The periodic acid-Schiff stain highlighted dermal blood vessels, and the direct immunofluorescence staining revealed faint C3 staining in some of the dermal blood vessels. The pathologist reported the final result as epidermal erythema multiforme or SJS with an explanation that the lack of inflammatory infiltrate made the histopathology consistent with SJS. The patient was eventually stabilized and discharged three months after his initial presentation. 

## Discussion

While the pathologist read the biopsy as SJS versus erythema multiforme, they were not provided with the context of methotrexate ingestion. Biopsies in cases of suspected methotrexate toxicity have shown reactive epidermal hyperplasia, dermal eosinophilia, ulcerated areas with a fibrin-covered base, keratinocyte necrosis, and negative immunofluorescence [5,6]. However, in SJS/TEN, supportive histopathologic features are full-thickness epidermal necrosis, sparse dermal inflammation, and orthokeratosis of the stratum corneum [1]. The medical team’s clinical diagnosis was methotrexate epidermal necrosis (MEN). Still, SJS caused by the IV vancomycin and piperacillin-tazobactam cannot be completely ruled out as the patient did receive one dose of etanercept, which would have been sufficient to treat SJS [7]. Clindamycin as a cause of SJS is significantly rarer. One important lab value that would help distinguish SJS and MEN is the number of T cells since SJS is a T-cell-mediated response that would not have occurred in the setting of severe leukopenia, as seen in this patient. However, it is unclear exactly when the patient became leukopenic, and therefore we still cannot definitively rule out SJS. Additionally, the methotrexate level was unexpectedly low, decreasing suspicion for MEN. The patient’s anemia, thrombocytopenia, relative eosinophilia, and SCID, however, are best explained by methotrexate toxicity.

## Conclusions

In this case, the patient was treated for all differential diagnoses given the severity of his condition, and treatments were appropriately updated as the team received more information from the patient and diagnostic tests. This unique case illustrates the importance of immediately checking a WBC count along with collection of a thorough history, medication reconciliation, and appropriate workup to rule out other causes for patients on methotrexate presenting with desquamating lesions. Comparing more cases of MEN to SJS and erythema multiforme to discover differences in histological presentation would decrease discrepancies in the clinical diagnosis since these entities present similarly in their symptoms and histology. It would also prevent unnecessary interventions since the prognosis and treatment of these entities varies significantly.
